# Implications and Recommendations for Research, Education, and Practice

**DOI:** 10.1093/geroni/igaa057.3023

**Published:** 2020-12-16

**Authors:** Janet Bettger, Susan Hughes, Mina Raj, Jaime Hughes

**Affiliations:** 1 Duke University School of Medicine, Durham, North Carolina, United States; 2 University of Illinois at Chicago, Chicago, Illinois, United States; 3 University of Michigan, Ann Arbor, Michigan, United States

## Abstract

Throughout this symposium, we have advocated for increased attention to how the chronicity and complexity of older adults’ health might impact their ability to maintain health behaviors over time. This final presentation will explore some implications of this chronicity and complexity on research study designs, formal education and professional training, and routine clinical practice. For example, existing research designs and funding mechanisms such as tightly controlled randomized controlled trials within a five-year grant may not permit researchers to study behaviors over an extended period of time and to do so within the real-world environments and social contexts in which older adults live. In contrast, integrating principles of community-engaged and participatory research, adaptive designs, pragmatic trials, and implementation science may encourage researchers to design for widespread dissemination. This session will close with recommendations to enhance educational initiatives and professional development to promote cross-disciplinary teams.

